# Treadmill exercise enhances therapeutic potency of transplanted bone mesenchymal stem cells in cerebral ischemic rats via anti-apoptotic effects

**DOI:** 10.1186/s12868-015-0196-9

**Published:** 2015-09-05

**Authors:** Yi-Xian Zhang, Ming-Zhou Yuan, Lin Cheng, Long-Zai Lin, Hou-Wei Du, Rong-Hua Chen, Nan Liu

**Affiliations:** Department of Rehabilitation, Union Hospital, Fujian Medical University, Fuzhou, 350001 People’s Republic of China; College of Integrative Medicine, Fujian University of Traditional Chinese Medicine, Fuzhou, 350122 People’s Republic of China; Department of Neurology, Union Hospital, Fujian Medical University, Fuzhou, 350001 People’s Republic of China

**Keywords:** MSCs, Stroke, Exercise, Apoptosis, Survivin, bcl-2

## Abstract

**Background:**

The transplantation of bone marrow stromal cells (MSCs) has proved to ameliorate ischemic brain injury in animals, but most transplanted MSCs undergo apoptosis in the ischemic penumbra, greatly compromising the therapeutic value of this treatment. Meanwhile, cell apoptosis can be inhibited by post-ischemia exercise which has been demonstrated to improve the expression of related anti-apoptotic proteins. The present study investigated whether treadmill exercise enhances the neuroprotective effects of transplanted MSCs in a rat experimental stroke model.

**Result:**

Rats were subjected to 2-h middle cerebral artery occlusion (MCAO). Twenty-four hours after reperfusion, they were assigned randomly to receive no MSCs treatment and no exercise (control group), intravenous transplantation of MSCs and treadmill exercise (MSCs + Ex group), MSCs transplantation only (MSCs group) and treadmill exercise only (Ex group). Neurological assessment, TUNEL staining and western blot were performed. Compared with the MSCs group and Ex group, the MSCs + Ex group reported markedly improved neurological function, significantly decreased apoptotic cells, and increased expressions of survivin and bcl-2 (*p* < 0.05 or *p* < 0.01, respectively). Interestingly, the treadmill exercise significantly inhibited the apoptosis of transplanted MSCs. As a result, the number of engrafted MSCs in the MSCs + Ex group was significantly higher than that in the MSC group (*p* < 0.01).

**Conclusions:**

Treadmill exercise enhances the therapeutic potency of MSCs by improving neurological function and possibly inhibiting the apoptosis of neuron cells and transplanted MSCs. These effects may involve an increased expression of survivin and bcl-2.

## Background

Stroke has been emerging as one of the most common causes of mortality and morbidity in modern society. Most stroke survivors live with residual impairments that diminish independence and quality of life [1]. Of its potential therapeutic candidates, bone marrow stromal cells (MSCs) transplantation has been previously demonstrated to ameliorate ischemic brain injury [2–8]. Although the exact mechanism is still unknown, it is suggested that the effect of MSCs transplantation depends on the number of transplanted MSCs [9]. However, the viability of MSCs is relatively poor when transplanted into the ischemic tissue [10]. On the other hand, much evidence suggests that exercise is beneficial for the recovery of brain function in animals and humans, and has been recommended as a therapeutic strategy for stroke. The beneficial effects of exercise therapy include moderation of neuronal damage, reduction of infarct volume, promotion of brain neurogenesis and angiogenesis, enhancement of brain metabolic capacity and brain plasticity [11–14]. Of particular importance, exercise can inhibit cell apoptosis by improving the expression of related anti-apoptotic proteins in the cerebral ischemic area [15]. Considering the anti-apoptotic effects of exercise, it may have synergetic neuroprotective effects with MSCs transplantation in the stroke treatment. Thus, the purpose of this study was to investigate whether treadmill exercise can enhance the neuroprotective effects of transplanted MSCs in a rat experimental stroke model by comparing the combined therapy of treadmill exercise and MSCs transplantation with either exercise or MSCs transplantation only.

## Methods

### Animal ethics

All experimental protocols were approved by the Institutional Animal Care and Use Committee of Fujian Medical University and were conducted in accordance with the National Institute of Health Guide for the Care and Use of Laboratory Animals (NIH Publications No. 80-23, revised in 1996). The investigators responsible for molecular, histological and functional studies were blinded to the treatment groups.

### Isolation, expansion and identification of MSCs

MSCs were isolated from rat bone marrow and expanded in vivo according to the method of Friedenstein et al. [16]. In brief, we euthanized Sprague–Dawley (SD) rats (weighted 80–100 g) to obtain the bone marrow. The harvested bone marrow cells were introduced into 100-mm dishes and cultured in a complete medium which consists of Dulbecco’s Modified Eagle’s Medium (DMEM, Sigma, USA) containing 10 % fetal bovine serum and antibiotics: 100 U/ml penicillin G, 100 mg/ml streptomycin, and 0.25 mg amphotericin B. The culture medium was replaced every 3 days and floating cells were discarded. The attached cells were divided into three new flasks after two passages and cultured until the cell density of the colonies grew to an approximately 90 % confluence. The cellular characteristics were identified by flow cytometric analysis. After blocked for nonspecific binding with buffer containing 1 % bovine serum albumin, the cells were incubated at 4 °C for 20 min with the following antibodies: anti-CD29, Phycoerythrin (PE), anti-CD106, PE, (Biolegend, USA), anti-CD44, luorescein isothiocyanate (FITC), anti-CD14, FITC and anti-CD45, FITC (AbD Serotec, USA). The matched isotype controls were purchased from AbD Serotec and Biolegend. At least 1 × 10^4^ cells per sample were acquired and analyzed.

### Differentiation assay of MSCs

The differentiation of MSCs in vitro towards the adipogenic and the osteogenic lineages were assayed as previously described [17, 18]. Briefly, for adipocyte differentiation, MSCs were cultured for 3 weeks with adipogenic medium, containing 10^−6^ M dexamethasone, 10 μg/ml insulin and 100 μg/ml 3-isobutyl-1-methylxantine (Sigma, USA); for osteoblast differentiation, MSCs was cultured for 3 weeks with osteogenic medium, containing 10^−7^M dexamethasone, 50 μg/ml ascorbic acid and 10 mM β-glycerophosphate (Sigma, USA). Oil-red-O and von kossa dyes were respectively employed to identify adipocytes and osteoblasts.

### Animal model

Adult male Sprague–Dawley rats (weighed 220–250 g) were used in this study. A middle cerebral artery occlusion (MCAO) was established with the modified Longa method [19]. Rats were initially anesthetized with isoflurane (containing 3 % induction, 1.5 % maintenance in 30 % O_2_ and 70 % N_2_O). The right common carotid artery, external carotid artery (ECA), and internal carotid artery were exposed. A 3.0 monofilament nylon suture (18.5 mm, determined by animal weight), with its tip rounded by heating it near a flame, was advanced from the ECA into the lumen of the internal carotid artery until it blocked the origin of the middle cerebral artery (MCA). Two hours after MCAO, animals were reanesthetized with isoflurane, and reperfusion was performed by withdrawal the suture until the tip cleared the lumen of the ECA. The rectal temperature was controlled at 37 °C with a feedback-regulated water heating system during the surgical procedure. Immediately after the procedure, rats were randomly divided into 4 groups: (1) control group, receiving no MSCs treatment and no exercise (n = 12), (2) Ex group, receiving exercise treatment only (n = 12), (3) MSCs group, receiving MSCs treatment only (n = 18), (4) MSCs + Ex group, receiving MSCs treatment and exercise (n = 18).

### Cell labeling and transplantation

MSCs were labeled with the lipophilic fluorochrome chloromethylbenzamido dialkylcarbocyanine (CM-DiI, Molecular Probes, USA) which was dissolved in dimethyl sulfoxide that had been incubated at 37 °C for 5 min and at 4 °C for 15 min (with a density of 1 ug CM-DiI per million cells) and then washed twice. After 2-h middle cerebral artery occlusion (MCAO) and 24-h reperfusion, rats in the MSCs and MSCs + Ex group were injected with 1 ml of phosphate-buffered saline (PBS) containing 3 × 10^6^ MSCs labeled with CM-DiI via the tail vein. Rats in the control group were injected with a comparable volume of PBS.

### Treadmill exercise training

A motor-driven treadmill (Treadmill Simplex II, Columbus Instruments, Columbus, OH, USA) was used in the study, which forces the rats to run via an electric stimulation system installed on the rear floor. Prior to the surgery, all rats underwent a 3-day treadmill exercise adaptation. Only the rats that learned to run were included in the study. A gradual treadmill exercise schedule was applied in which all qualified rats ran at 4 m/min for the first day, 8 m/min for the second day, and then 12 m/min for the remaining days with an inclination of 0°. The rats in the Ex group and MSCs + Ex group underwent the treadmill exercise 2 days after the surgery at an intensity of 30 min per day for a maximum of 14 days. The rats in the control and MSCs groups were allowed to move freely in their cages, but no additional treadmill exercise was employed.

### Behavioral testing

All rats underwent behavioral testing at day 1 and 14 after MCAO using a modified neurological severity score, as described previously [9]. In brief, this score was a composition of the motor (response to raising the rat by the tail or placing the rat on a flat surface), sense (response to visual, tactile, and proprioceptive stimulation), balance (response to placement and posture on a narrow beam and time before dropping), and reflex (response of pinna, cornea and startle) tests. This compositive neurological function test was graded on a scale of 0–18 (normal score, 0; maximal deficit score, 18), where one point was awarded for the inability to perform a task or for a lack of a tested reflex.

### Assessment of apoptosis in ischemic penumbra

The anti-apoptotic effects of each treatment were examined at 14 days after MCAO (n = 6 in each group). Paraffin embedded sections were prepared for TUNEL assay. TUNEL staining was performed with a commercially available kit (ApopTag Plus; Serological Corporation, USA). The number of TUNEL-positive cells per field in the ischemic penumbra was counted and expressed as the average in the 6 fields. To evaluate apoptosis of transplanted MSCs in the ischemic penumbra, 12 additional rats were used (MSCs group, n = 6; MSCs + Ex group, n = 6). At 14 days after MCAO, rats were transcardially perfused with heparinized saline, followed by 4 % paraformaldehyde, and then 8 μm-thick coronal frozen sections were cut on a cryostat frozen sections. TUNEL staining was performed with a commercially available kit (ApopTag Fluorescein kit, USA). The number of TUNEL-and CM-DiI-positive cells was counted and expressed as the average in the 6 sections.

### Western Blot for survivin and bcl-2 expression in ischemic penumbra

Rats were euthanized with isoflurane 14 days after MCAO (n = 6 in each group). The protein concentration from the ischemic penumbra was determined using the bicinchoninic acid (BCA) protein assay kits (Beyotime Biotechnology, P. R. China). Thirty micrograms denatured protein were separated by SDS-PAGE and loaded on 10 % acrylamide gel for electrophoresis and transferred onto a polyvinylidene difluoride membrane (PVDF, Invitrogen, USA). The membranes were incubated with rabbit anti-survivin (1:500, Santa Cruz Biotechnology Inc., Santa Cruz, CA, USA) and rabbit anti-Bcl-2 (1:500, Santa Cruz Biotechnology Inc., Santa Cruz, CA, USA), respectively. Horseradish-peroxidase-conjugated goat-anti-mouse IgG or goat anti-rabbit IgG (1:1000, Bio-Rad Laboratories) was used as the secondary antibody and GADPH was used as a loading control. All bands from western blot were analyzed using Image J software (version 1.6 NIH) to verify the relative level of survivin and bcl-2 defined as the optical density ration of survivin or bcl-2 over GADPH.

### Statistical analysis

Data were presented as mean values and standard deviation. Student’s *t* test was used for two-group comparisons and one-way ANOVA (analysis of variance) with Scheffe’s post hoc test was used to identify differences among all groups. A P value of less than 0.05 was considered as statistically significant.

## Results

### Characterization, differentiation and labeling of the cultured MSCs

**Fig. 1:**
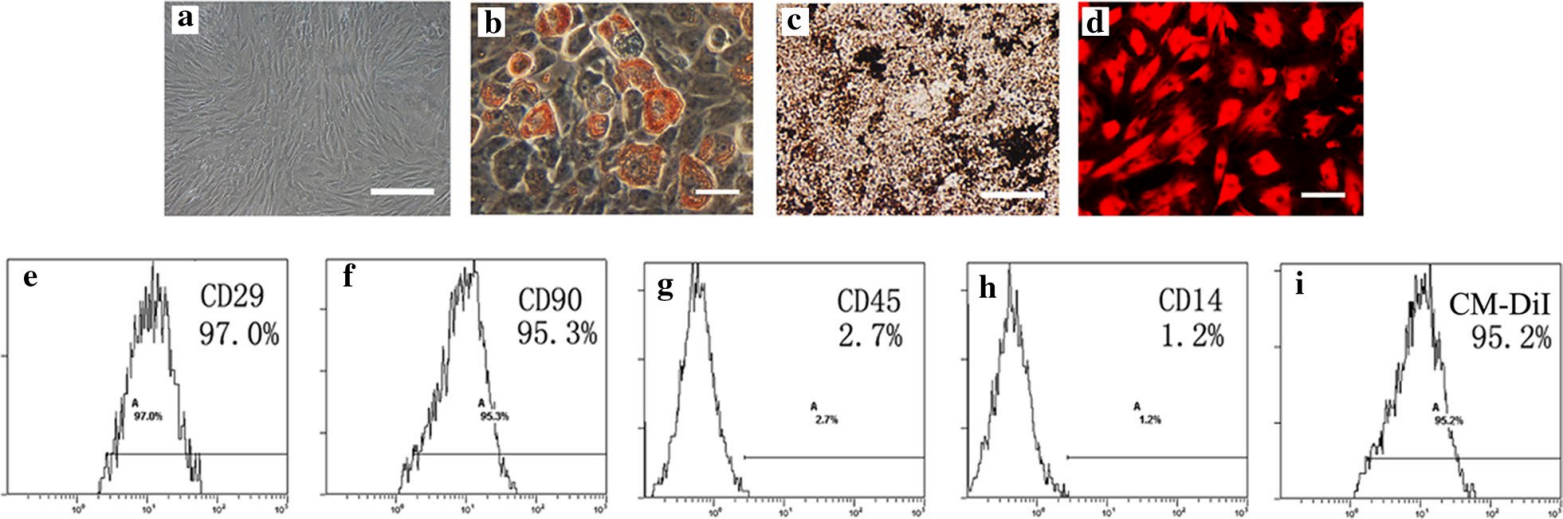
Characterization, differentiation and labeling of MSCs: **a** The cultured cells displayed the typical “fibroblast-like” morphology, **b** adipocyte differentiation of MSCs: upon induction with adipocyte induction media, cells showed adipocyte globules on oil red “O” staining, **c** osteogenic differentiation of MSCs: upon induction with osteogenic induction media, cells showed calcium deposites on von kossa staining, **d** MSCs were labeled with CM-DiI, **e**–**h** flow cytometry analysis: MSCs expressed the markers molecules CD29, CD90 and negative for the blood cell surface molecules CD45, CD14. The percentage of positivity was mentioned in the *brackets*, **i** flow cytometry analysis showed that more than 95 % MSCs were labeled with CM-DiI (*scale bar* 200um in **a** and **c**, 50 μm in **b** and **d**)

At day 3 after planting, the culture cells were observed to scatter in a manner of colonial distributions. Then, at days 8–9, the dish was covered with long-spindle cells. The passage cells were uniformly distributed and displayed the typical “broblast-like” morphology (Fig. 1a). Flow cytometric analysis showed that the 3rd passage MSCs highly expressed the surface marker molecules CD29 (97.0 %), CD90 (95.2 %) and lowly expressed the blood cell surface molecules CD14 (1.2 %) and CD45 (2.7 %) (Fig. 1d–h). After 3 weeks of adipogenic induction and oesteogenic induction, the cells displayed lipid-laden adipocyte phenotype by Oil red ‘O’ dyes and calcium-deposited osteoblast phenotype by von kossa staining, respectively (Fig. 1b, c). Flow cytometric analysis showed more than 95 % MSCs were successfully labeled by CM-DiI.

### Behavioral testing

**Fig. 2:**
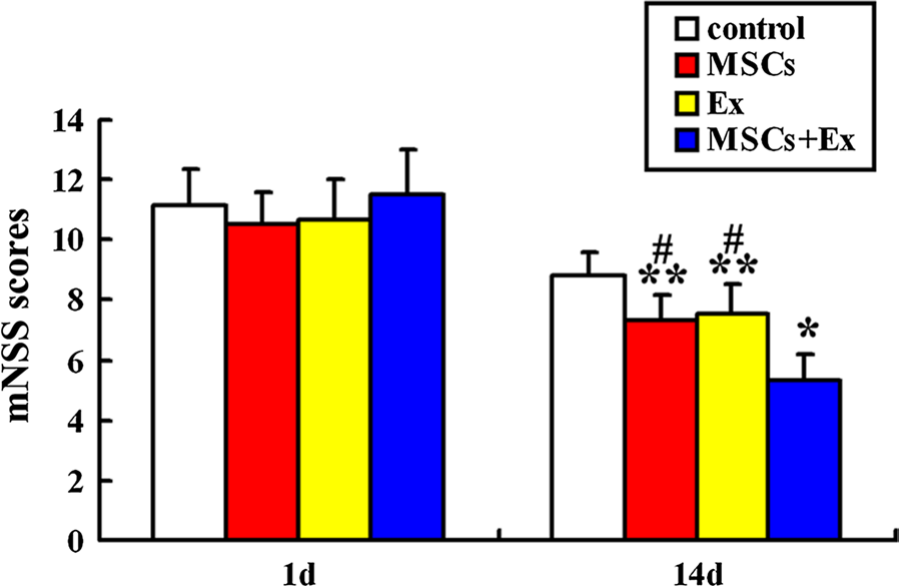
Neurological scores: The score of mNSS at day 1 and 14 after MCAO in the control group, MSCs, Ex, and MSCs + Ex groups. Data are expressed as the mean ± SD (n = 6). **p < 0.05, as compared with control group, *p < 0.01, as compared with control group, ^#^ *p* < 0.01, as compared with MSCs + Ex group

There was no significant difference in neurological severity scores among the 4 groups at day 1 after MCAO. At day 14, scores of the MSCs group and Ex group were lower than those of the control group (7.33 ± 0.82 vs 8.83 ± 0.75, *p* < 0.05; 7.50 ± 1.37 vs 8.83 ± 0.75, *p* < 0.05, respectively) and no significant difference was reported between the MSCs and Ex groups. Interestingly, the scores in MSCs + Ex group were much lower than those of MSCs group and Ex group (5.33 ± 0.82 vs 7.33 ± 0.82, *p* < 0.01; 5.33 ± 0.82 vs 7.50 ± 1.37, *p* < 0.01, respectively) (Fig. 2).

### MSCs transplantation combined with exercise induces a greater anti-apoptotic effect on neuronal cells

**Fig. 3:**
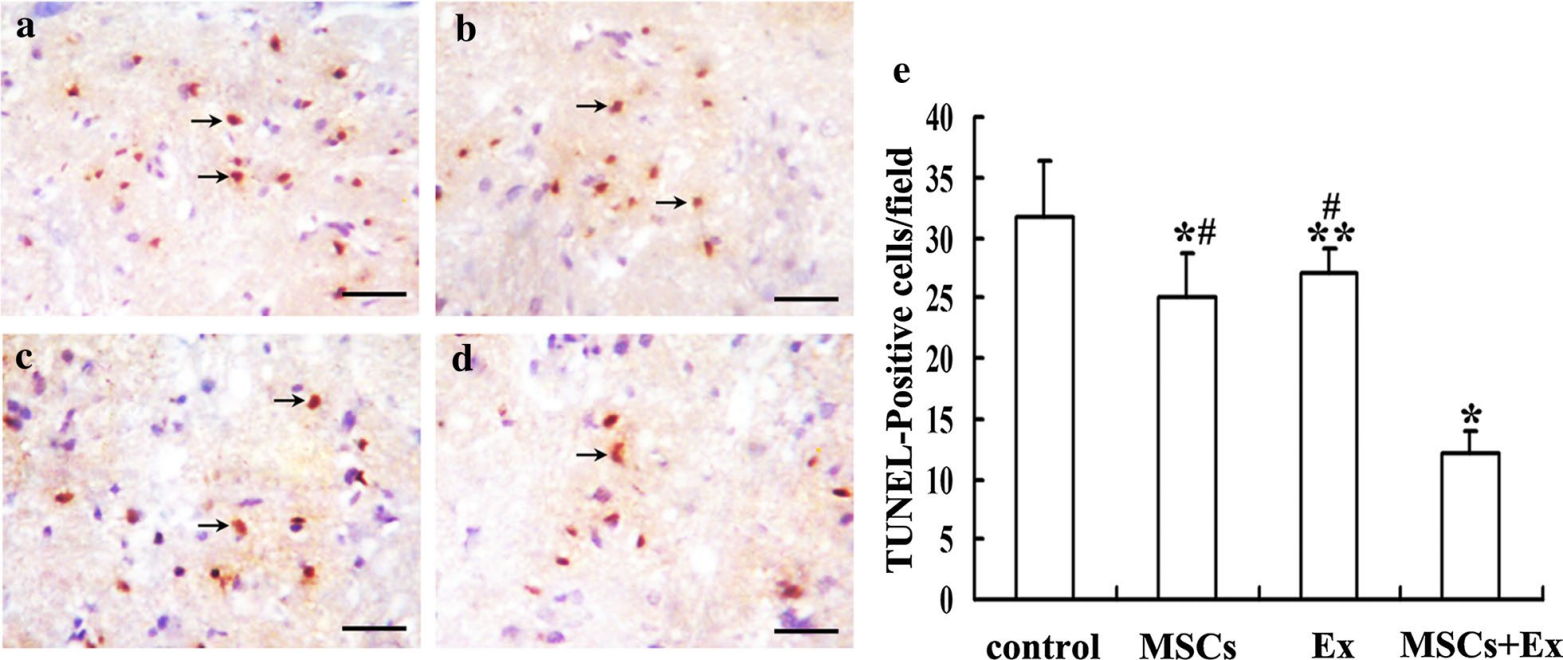
Detection of cell apoptosis in the ischemic penumbra: **a**–**d** representative of photomicrographs of TUNEL staining in the ischemic penumbra 14 days after MCAO. The number of TUNEL staining-positive cells in the MSCs + Ex group (*black arrows*, DAB, *brown*) was markedly the lowest among the 4 groups. **a** control group, **b** MSCs group, **c** Ex group, **d** MSCs + Ex group. **e** Quantitative analysis of the number of TUNEL-positive cells. Data are expressed as the mean ± SD (n = 6). **p < 0.05, as compared with control group, *p < 0.01, as compared with control group, ^#^ *p* < 0.01 as compared with MSCs + Ex group

Ischemia induced significant TUNEL-positive cells in the ischemic penumbra at day 14 after MCAO (Fig. 3a). Quantitative analysis demonstrated that the number of TUNEL-positive cells in the MSCs and Ex group was lower than that in the control group (25.00 ± 3.74 vs 31.67 ± 4.63, *p* < 0.01; 27.00 ± 2.00 vs 31.67 ± 4.63, *p* < 0.05, respectively) and that no significant difference was found between the MSCs and Ex groups. Of note, the number of TUNEL-positive cells in the MSCs + Ex group was significantly lower than that of the MSCs and Ex groups (12.17 ± 1.72 vs 25.00 ± 3.74, *p* < 0.01; 12.17 ± 1.72 vs 27.00 ± 2.00, *p* < 0.01, respectively) (Fig. 3b, c).

### MSCs transplantation combined with exercise induces a greater expression of survivin and bcl-2

**Fig. 4:**
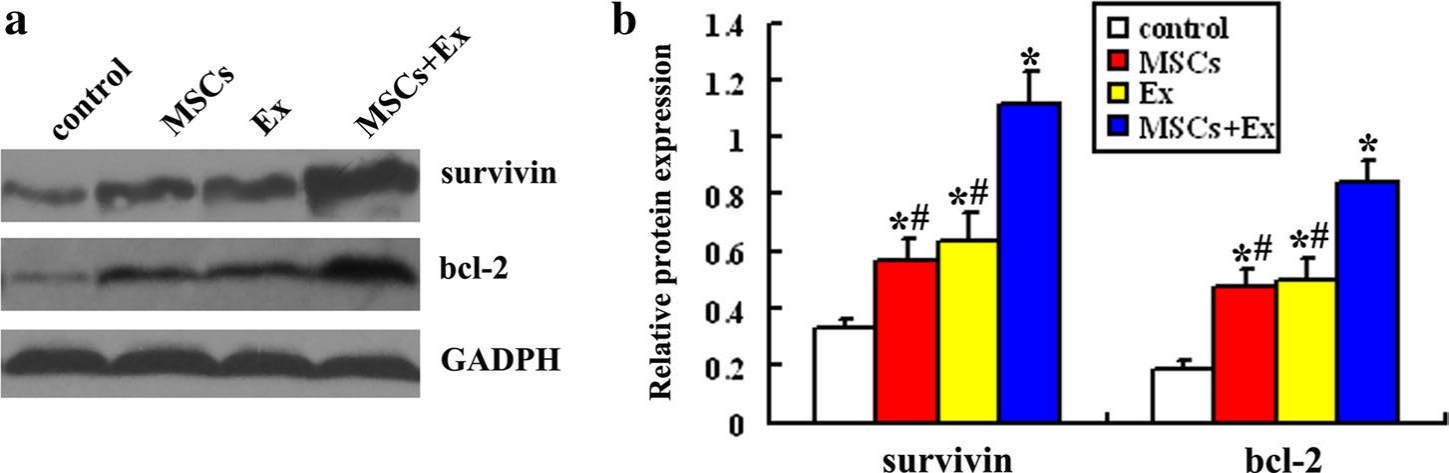
Survivin and bcl-2 expression in the ischemic penumbra: **a** representative photomicrographs of protein bands of survivin and bcl-2 in the ischemic penumbra 14 days after MCAO. **b** Semi-quantitative results for survivin and bcl-2. Data are expressed as the mean ± SD (n = 6). *p < 0.01, as compared with control group. ^#^ *p* < 0.01, as compared with MSCs + Ex group

To determine whether survivin and bcl-2 are involved in the anti-apoptotic effects, we examined the level of protein expression in the ischemic penumbra by western blot 14 days after MCAO (Fig. 4). The result demonstrated that compared with the control group, the level of survivin was higher in the MSCs (0.57 ± 0.08 vs 0.33 ± 0.04, *p* < 0.01) and Ex group (0.64 ± 0.10 vs 0.33 ± 0.04, *p* < 0.01) and that there was no significant difference between the MSCs and Ex groups. The level of survivin expression in the MSCs + Ex group was significantly higher than that of the MSCs and Ex groups (1.12 ± 0.11 vs 0.57 ± 0.08, *p* < 0.01; 1.12 ± 0.11 vs 0.64 ± 0.10, *p* < 0.01, respectively). Similarly, the level of bcl-2 expression was higher in the MSCs group (0.47 ± 0.07 vs 0.19 ± 0.04, *p* < 0.01) and Ex group (0.49 ± 0.08 vs 0.19 ± 0.04, *p* < 0.01) when compared with that of the control group; there was no significant difference between the MSCs and Ex groups. The level of bcl-2 expression in the MSCs + Ex group was significantly higher than that of the MSCs and Ex groups (0.84 ± 0.08 vs 0.47 ± 0.07, *p* < 0.01; 0.84 ± 0.08 vs 0.49 ± 0.08, *p* < 0.01, respectively).

### Exercise inhibits the apoptosis of transplanted MSCs

**Fig. 5:**
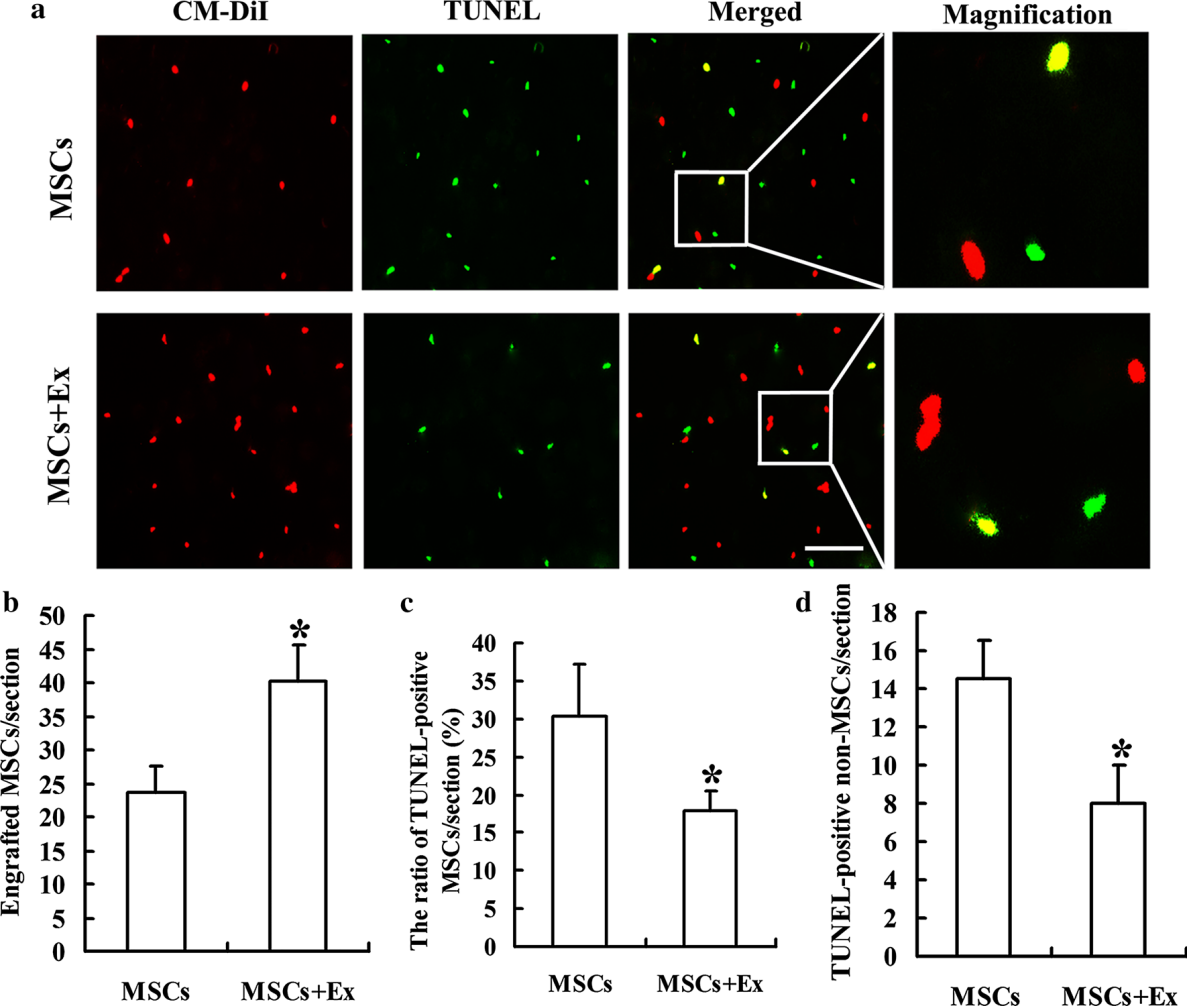
Detection of engrafted MSCs apoptosis in the ischemic penumbra: **a** representative photomicrographs of MSCs apoptosis in the ischemic penumbra at day 14. Transplanted MSCs were labeled with CM-DiI (*red*). TUNEL staining was used to detect apoptotic cells (*green*). TUNEL-positive MSCs (double-positive cells merged, *write arrow*) were frequently observed. **b** Quantitative analysis of the number of engrafted MSCs. **c** Quantitative analysis of the ratio of TUNEL-positive MSCs, **d** quantitative analysis of the number of TUNEL-positive non-MSCs. Data are expressed as the mean ± SD (n = 6). *p < 0.01, as compared with control MSCs group. *Scale bars* 100 μm

The CM-DiI-labeled MSCs were detected in the ischemic penumbra after transplantation (Fig. 5a). Quantitative analysis indicated that 14 days after MCAO, the number of engrafted MSCs in the MSCs + Ex group was sharply higher than that in the MSCs group (40.17 ± 5.46 vs 23.67 ± 3.88, *p* < 0.01) (Fig. 5b). The ratio of TUNEL-positive MSCs in the MSCs + Ex group was markedly lower than that in the MSCs group (19.84 ± 2.7 % vs 30.32 ± 6.90 %, *p* < 0.01) (Fig. 5c). Meanwhile, the number of TUNEL-positive non MSCs, including neuronal cells, was also decreased by treadmill exercise. Quantitative analysis indicated that the number of TUNEL-positive non MSCs in the MSCs + Ex group was sharply lower than that in the MSCs group (18.0 ± 1.41 vs 4.5 ± 1.52, *p* < 0.01) (Fig. 5d).

## Discussion

In the present study, we showed that: (1) Treadmill exercise and MSCs transplantation inhibited the apoptosis in the ischemic penumbra; (2) Only a few transplanted MSCs were detected in the ischemic penumbra of the MSCs group, while the combination of treadmill exercise and MSCs transplantation increased the number of engrafted MSCs; (3) The combination of treadmill exercise and MSCs transplantation up-regulated the expression of survivin and bcl-2, enhancing the anti-apoptosis effects on neuron cells in the ischemic penumbra, and finally greatly improved the recovery of neurological function when compared with treadmill exercise or MSCs transplantation only.

Previous studies have suggested that pre- or post-ischemic exercise reduces brain injury and improves neurological deficits in a rat stroke model [14, 20]. The present study demonstrated that early post-ischemic treadmill exercise improved neurological functions in rats. However, the underlying mechanisms still remain unclear. It is well known that an ischemic insult to the brain can result in neural loss due to either necrosis or apoptosis or both. Unlike most cells in the ischemic core, which undergo irreversibly necrosis, many cells in the ischemic penumbra will undergo apoptosis. Thus the cells in the ischemic penumbra are potentially recoverable and there is an opportunity for salvage. Expectedly, in the present study, treadmill exercise decreased TUNEL-positive cells in the ischemic penumbra 14 days after stroke. These results suggest that treadmill exercise improves neurological functions, at least in part, through the inhibition of cell apoptosis in the ischemic penumbra.

Recently, MSCs transplantation has been shown to play beneficial roles in repairing the damaged brain tissue and improving functional outcome in experimental rodent models of focal ischemia. In this study, our results confirmed that transplantation of MSCs 24 h after ischemia induced significant improvement of functional deficit in adult rats. This finding is consistent with those of other studies showing functional improvement induced by intravenously transplanted MSCs [9]. Furthermore, we examined the apoptotic cells in the brain using TUNEL staining. Our results showed that the TUNEL-positive cells in the ischemic penumbra were down-regulated after MSCs transplantation, which suggests that the neuroprotective effect of MSCs is related not only to their role in neurogenesis, angiogenesis, alleviating inflammatory cascade reaction and formatting of structural reorganization, as previously reported [3, 4, 6, 21], but also to their potential anti-apoptotic effect.

The molecular mechanisms of apoptosis have been thoroughly studied and are currently well-understood. Survivin and bcl-2, the family of proto-oncogenes, have been documented to regulate apoptosis. Survivin is expressed in a cell cycle-regulated manner and localized together with caspase 3 and 7 on microtubules within centrosomes to suppress apoptosis in the G2/M phases [22]. Unlike survivin, bcl-2 suppresses apoptosis by regulating the release of cytochrome c [23], which is often considered as a point of no return in the subsequent caspase cascade. It has been suggested that the over-expression of survivin or bcl-2 protects neurons against cerebral ischemia [23–25]. In addition, several studies have shown that exercise or MSCs transplantation inhibits the apoptosis of neurons through inducing the expression of survivin or bcl-2 or both in a model of stroke [15, 26]. In present study, we used a method of western blot to detect the level of survivin and bcl-2 expression. We found that either treadmill exercise or MSCs transplantation after cerebral ischemia promoted survivin and bcl-2 expression in the ischemic penumbra at day 14, suggesting that these proteins suppress apoptosis in the ischemic lesion. Interestingly, MSCs transplantation plus exercise induced a greater expression of surivin and bcl-2 than exercise or MSCs transplantation only. The result is consistent with the finding that MSCs transplantation plus exercise greatly decreased the number of TUNEL-positive cells in the ischemic penumbra, suggesting that a combination of exercise with MSCs transplantation has synergetic inhibitive effects on neuronal apoptosis.

CM-DiI dye is a carbocyanine membrane probe with strong photo-stable fluorescence, long cellular retention, and minimal cytotoxicity [27]. Moreover, DiI is retained in cells throughout fixation, permeabilization, and paraffin-embedding procedures [28]. Previous studies have demonstrated that CM-DiI is useful for long-term MSCs labeling [29, 30]. In the present study, we pre-labeled MSCs with CM-DiI dye. Only a few transplanted MSCs were detected in the ischemic penumbra of the MSCs group. However, a lot more CM-DiI labeled MSCs were detected in the MSCs + Ex group, suggesting that exercise contributes to prolonging the viability of transplanted MSCs. Meanwhile, the ratio of TUNEL-positive MSCs in the MSCs + Ex group was sharply lower than that in the MSCs group. These results suggest that treadmill exercise contributes to inhibiting the apoptosis of transplanted MSCs and finally prolonging the survival time of transplanted MSCs. Moreover, the number of apoptotic non-MSCs in the MSCs + Ex group was sharply lower than that in the MSCs group, suggesting direct protective effects of treadmill exercise on the ischemic penumbra. Finally, we observed that a combination of treadmill exercise and MSCs transplantation markedly improved neurological functions compared with MSCs transplantation only. Considering the anti-apoptotic effects of treadmill exercise and MSCs transplantation, treadmill exercise may have potential synergetic neuroprotective effects and enhance the therapeutic potency of MSCs transplantation, leading to further improvement in neurological functions after stroke.

## Conclusions

Treadmill exercise enhances the therapeutic potency of MSCs by improving neurological function and possibly through inhibition of cells apoptosis, including neuron cells and transplanted MSCs themselves. These effects may involve a greater expression of survivin and bcl-2. A combination of treadmill exercise and MSCs transplantation may be a new therapeutic strategy for the treatment of cerebral ischemia.
